# A compiled and systematic reference map of nucleosome positions across the *Saccharomyces cerevisiae *genome

**DOI:** 10.1186/gb-2009-10-10-r109

**Published:** 2009-10-08

**Authors:** Cizhong Jiang, B Franklin Pugh

**Affiliations:** 1 Center for Eukaryotic Gene Regulation, 456 North Frear Laboratory, Department of Biochemistry and Molecular Biology, The Pennsylvania State University, University Park, PA 16802, USA; 2 Current address: The School of Life Sciences and Technology, Tongji University, Shanghai, 200065, PR China

## Abstract

Nucleosomes have position-specific functions in controlling gene expression. A complete systematic genome-wide reference map of absolute and relative nucleosome positions is needed to minimize potential confusion when referring to the function of individual nucleosomes (or nucleosome-free regions) across datasets. We compiled six high-resolution genome-wide maps of *Saccharomyces cerevisiae *nucleosome positions from multiple labs and detection platforms, and report new insights. Data downloads, reference position assignment software, queries, and a visualization browser are available online http://atlas.bx.psu.edu/.

## Abstract 2

Different  genome-wide reference maps of Saccharomyces cerevisiae nucleosome positions are compiled and can be visualized on a browser.

## Rationale

Eukaryotic chromatin exists as a repeating unit of nucleosome particles [1,2], where approximately 147 bp of DNA coils around a histone octamer [3,4]. Nucleosome positioning makes the underlying DNA accessible or refractory. As a result, nucleosomes can regulate processes that require access to DNA, such as DNA replication and transcription [5]. In addition, many gene regulatory proteins interact with nucleosomes [6]. Thus, the determination of nucleosome positions is key to understanding genome access and how the transcription machinery functions *in vivo*. Moreover, as we learn more about distinct functional roles of individual nucleosome positions, it is critical that nucleosome positions be unambiguously identified in different studies.

An early study identified nucleosome positions primarily along chromosome III in *Saccharomyces cerevisiae *using a tiled microarray approach [7]. From this study, the concept that nucleosomes generally occupy fixed positions at genes took hold. High-resolution microarray approaches have now produced two complete maps of nucleosome positions in *S. cerevisiae *[8,9]. With the advances in high-throughput DNA sequencing technology, additional higher resolution genome-wide maps of nucleosome positions have now been completed using Roche/454 pyrosequencing [10-12] and the Illumina/Solexa 1G sequencer [13]. Genome-wide maps of nucleosome positions have also been produced in other species, such as in *Drosophila *using Roche/454 pyrosequencing [14], in *Caenorhabditis elegans *using the Applied Biosystems SOLiD sequencer [15], and in humans using the Illumina/Solexa 1G sequencer [16].

The genome-wide maps of nucleosome positions have shown that nucleosomes are highly phased near the 5' end of genes, and reside at a canonical distance from transcription start sites (TSSs) [8-16]. Individual nucleosomes may have distinct functions depending upon their context in and around genes. The +1 nucleosome (the first one downstream of the TSS) might present a barrier to transcription by RNA polymerase II in *Drosophila *[14]. In addition, precise positioning of the +1 nucleosome may cause precise positioning of downstream nucleosomes due to statistical principles of nucleosome packaging [2,11]. Another example of a potential position-specific function related to the +1, +2, and +3 nucleosomes may be their preferential methylation on lysine 4 of histone H3 (H3K4me3) [16].

Just upstream of the +1 nucleosomes often resides a nucleosome-free region (NFR) that coincides with the promoter region. Maintenance of this NFR may be due in part to poly dA:dT tracts (where homopolymeric adenylate is base-paired with homopolymeric thymidylate) in the promoter, which resist incorporation into nucleosomes, and in part by sequence-specific DNA binding proteins (for example, Reb1) that help position nucleosomes [17], and by the intrinsic tendency of underlying DNA sequences to wrap around the histone core octamer [7,11,12,18-20]. Such positioning may also be resisted by chromatin remodeling complexes, such as ISW2, which preferentially moves nucleosomes into the NFR of certain genes [9]. Nucleosomes may come and go at the different positions, including the NFR [13]. The -1 nucleosome sits where many *cis*-regulatory elements reside and thus has the potential to control assembly of the transcription machinery in a way that no other nucleosome position can.

With individual nucleosome positions having potentially distinct functions, it is important that studies utilize a common systematic nomenclature for identifying nucleosome positions, so as to maintain consistency in characterizing individual nucleosome function across datasets. Currently, there is no standard in calling individual nucleosome positions, even though several genome-wide maps of nucleosome positions have been published. For example, the nucleosome closest to the 3' end of a gene has been labeled as a -1 nucleosome [9], and the rare nucleosome that appears in the NFR region has been defined as the -1 nucleosome in humans [16]. In contrast, other studies have identified the -1 nucleosome as immediately upstream of the NFR [10,11,14]. Because these different conventions have the potential to cause confusion and conflicting interpretations, we sought to develop a convention for defining a nucleosome reference map in *Saccharomyces*. Such a convention would also be applicable to other organisms once sufficient maps have been obtained so as to derive a consensus reference.

In this study, we collected six sets (five published and one unpublished) of nucleosome positions across the genome of the conventional wild-type yeast strain S288C, under yeast peptone dextrose (YPD) media (rich) growth condition. The set of maps were generated from different laboratories using different technologies [8,9,11-13], including Affymetrix 1.0 tiling arrays, and Roche/454, Illumina/Solexa, and Applied Biosystems SOLiD genome sequencing platforms. Because some nucleosomes were not detected in all six datasets, the consensus of these six maps provides the most complete and accurate set of nucleosome positions. Each reference nucleosome was assigned a genomic coordinate to which nucleosomal positions from new datasets can be linked to (see Materials and methods). From this reference map, we categorized individual nucleosomes into -1, 0, +1, +2, +3, etc. positions relative to the TSS. We constructed a nucleosome retrieval system that allows users to extract nucleosome positions in a given region or from a list of genes. We also constructed an assignment system that assigns any nucleosome position in a dataset to a reference position. The reports provide the coordinates and relative position (for example, -1, 0, +1, etc.) of each nucleosome and its distance from the associated gene TSS. This reference map of nucleosome positions and its associated retrieval system should have broad applicability.

## Implementation

### A complete reference set of nucleosomes, arrays, linkers and nucleosome-free regions

**Figure 1:**
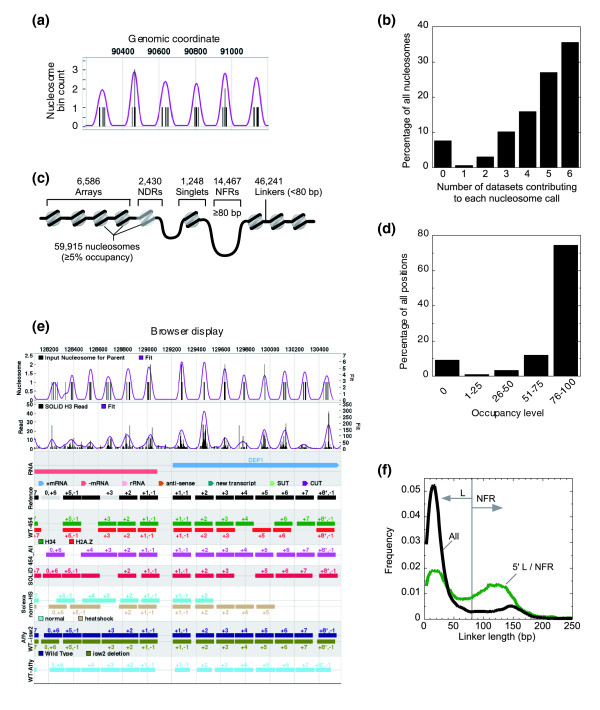
**A consensus of consensus nucleosome calls defines the nucleosome reference map**. **(a) **Screen shot of six consensus nucleosome calls (vertical bars) in which each is a consensus of positions from six datasets (five datasets for positions 1 and 4). Shown is chromosome 11 (loci 90200 to 91200). Narrower peaks have a stronger consensus. The trace indicates the probability landscape for a reference nucleosome. **(b) **Bar graph of the number of datasets contributing to the set of reference nucleosome positions (including hypothetical positions). **(c) **Illustration of the types of nucleosomes in the yeast genome, and their specifications. **(d) **Bar graph indicating nucleosome occupancy level throughout the genome at quartile intervals. **(e) **Browser screen shot of consensus nucleosome positions from 128000 to 130600 at chromosome 1. Any location can be queried online [21]. The top track, indicated as 'RNA', provides coordinates of different types of RNA transcripts as color-coded by the legend immediately under it. The 'Reference' track provides the location and the positional number of the reference nucleosome calls. The darkness of the box indicates the mode-normalized nucleosomal occupancy: light gray, < 5% (that is, in NFRs); intermediate gray, 5 to 50% (that is, in nucleosome-depleted regions); dark gray, 50 to 100%; black, 100%. The remaining six sets of tracks represent the individual consensus calls from datasets 1 to 6 (see Materials and methods). Within each set, additional nucleosome subsets are shown (for example, H2A.Z nucleosomes, nucleosomes from heat-shocked cells, and nucleosomes from an *isw2 *deletion strain). One nucleosome may have multiple names (for example, '+1,-1') when it is associated with more than one gene (exemplified in red boxes). Asterisks indicate this nucleosome is the terminal one to its associated gene (that is, the last one at the 3' end of the gene). **(f) **Smoothed frequency distribution of all linker lengths and only those found at the 5' end of genes.

We compiled the genomic nucleosome positions from five published datasets, and one published here (Table 1). Datasets 1 to 4 were produced by massively parallel DNA sequencing from three different platforms to map nucleosome positions, whereas datasets 5 and 6 use a microarray hybridization approach from two different platforms. The vast majority of nucleosome positions were found in most of the six nucleosomal datasets (Figure 1a, b). Rather than report occupancy levels as fold over background, as is common practice for chromatin immunoprecipitation (ChIP), but difficult to define when most of the genome is occupied, we scaled occupancy levels to range from 0 to 100% as described in the methods. This represents a new and more versatile means of reporting genome-wide occupancy levels.

**Table 1:** Nucleosome datasets as input to generate the consensus nucleosomes

Set number	Strain	Growth condition	Platform	Nucleosome count	Tag count
1	BY4741	YPD, 25°C	Roche GS20	54,753	1,206,057
2	BY4741	YPD, 30°C	Roche GS20	48,126	378,686
3	BY4741	YPD, 25°C	AB SOLiD	55,124	12,477,015
4	S288C	YPD, 30°C	Illumina Solexa 1G	49,043	514,803
5	S288C	YPD, 28°C	Affymetrix 1.0 (5 bp)	63,026	
6	BY4741	YPD, 30°C	Affymetrix custom	70,871	

References by set number: 1 [11]; 2 [12]; 3, this paper; 4 [13]; 5 [9]; 6 [8].

We identified 59,915 nucleosome positions (>90% of all possible positions) that had an occupancy level of ≥5% in *S. cerevisiae *grown in YPD media (Figure 1c; Additional data file 1). The degree of positional phasing/fuzziness of these nucleosomes varied from highly positioned to essentially randomly positioned, which we report in a quantifiable manner (Additional data file 1). Another 6,238 potential positions were sufficiently large to accommodate a nucleosome but had <5% occupancy (of which >95% had zero occupancy). The lack of detection was not due to insufficient coverage. For example, in dataset 3, which has not been previously published and contains over 12 million tags (ten times more than any other dataset), each measured nucleosome averages 160 tags. In contrast, the potential/hypothetical positions contained no tags (that is, the median value was zero), and were not detected in any of the six datasets. We ruled out the possibility that such regions are un-mappable due to technical reasons (for example, repeated regions). Thus, many accessible regions in the genome are truly nucleosome-free. Whether other proteins that are bound to these regions or the underlying DNA sequence exclude nucleosomes in such regions remains to be determined.

If we assume that the most frequently encountered nucleosome occupancy level (in terms of tag counts) corresponds to 100% occupancy, then >95% are present at least half the time (Figure 1d). Only approximately 4% of all detected nucleosomes were relatively depleted (designated as 5 to 50% occupancy level, and termed nucleosome-depleted regions). We identified 6,586 nucleosomal arrays (defined as two or more contiguous nucleosomes, with each having >50% occupancy level and linkers <146 bp) and 1,248 singlets (Additional data file 1).

A browser for graphically viewing the genomic distribution of reference nucleosomes as well as nucleosome calls from individual datasets can be queried or browsed online [21], in a format shown in Figure 1e. The browser also provides a means to observe changes in nucleosome positions (due to eviction, acquisition, or a shift, or discrepancies between datasets - for example, see Figure S2 in Additional data file 2) in a region of interest. Since the reference set of nucleosome positions represents a complete set of nucleosomes in yeast growing asynchronously in YPD media, one can identify missing nucleosomes in a test dataset.

If we define the region between the borders of adjacent nucleosomes as linkers, then the genome-wide distribution of linkers is bimodal (Figure 1f, black trace). The distribution of the major population is centered at 15 bp. The minor distribution is broadly distributed between approximately 100 and 200 bp, and is particularly enriched at the 5' ends of genes (green trace). The two peaks might represent distinct functions of linkers, the major peak being the most common distance between nucleosomes (15 bp), and the other being roughly the size of a displaced nucleosome. We therefore classified linkers into two groups, partitioned at the minimum in Figure 1f: 46,241 linkers having a length of 0 to 79 bp, and 14,467 NFRs that are 80 or more base-pairs in length. Thus, we report a systematic definition of an NFR.

### Precision of sequencing and hybridization platforms

**Figure 2:**
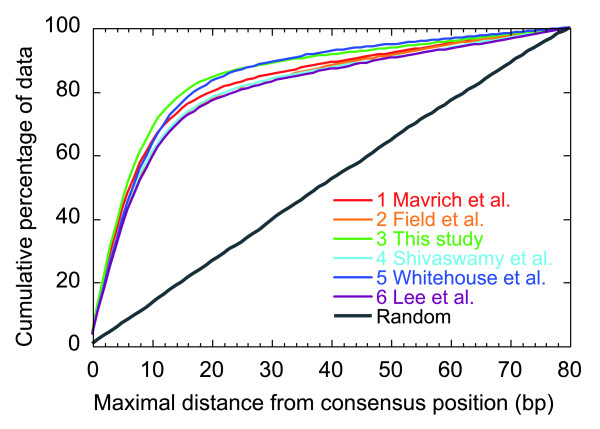
**Cumulative error associated with the six sets of input nucleosomes compared against the reference set**. The error interval is the midpoint distance between the reference nucleosome and the query nucleosome. Only those reference nucleosomes that were contributed by all six datasets were used in the error analysis. Each dataset is described in Table 1.

With the reference set in place we determined the precision of all nucleosome calls in each dataset relative to the reference set (Figure 2). For all data sets, the median precision was approximately 5 to 7 bp, compared to 38 bp for a randomized set. At one extreme, the SOLiD platform (dataset 3) called 80% of the nucleosomes within 16 bp of the reference positions, while at the other extreme the Affymetrix platform called 80% of the nucleosomes within 24 bp of the reference, compared to 64 bp for a randomized set. This difference may be due to any combination of differences in sample preparation, platform resolution, and bioinformatic peak calling. We further performed the same error analysis on individual nucleosome positions relative to the TSS, and found the Roche/454 platform (or its associated methodology) provided the highest precision (median precision of 4 bp, with 80% of the nucleosomes called within <10 bp of the reference position) at the +1 nucleosome position (Figure S3 in Additional data file 2). The relatively low error associated with +1 nucleosomes reflects their highly phased state. It is important to note, however, that many other nucleosome positions are not phased (having more fuzzy or delocalized positions), and so reference nucleosome positions at such positions are not particularly meaningful. Additional data file 1 reports the fuzziness of each nucleosome, and this should be taken into consideration when specifying nucleosome positions. For example, shifting of a delocalized nucleosome may not be meaningful or accurate.

### Nucleosome positioning around transcription start sites

**Figure 3:**
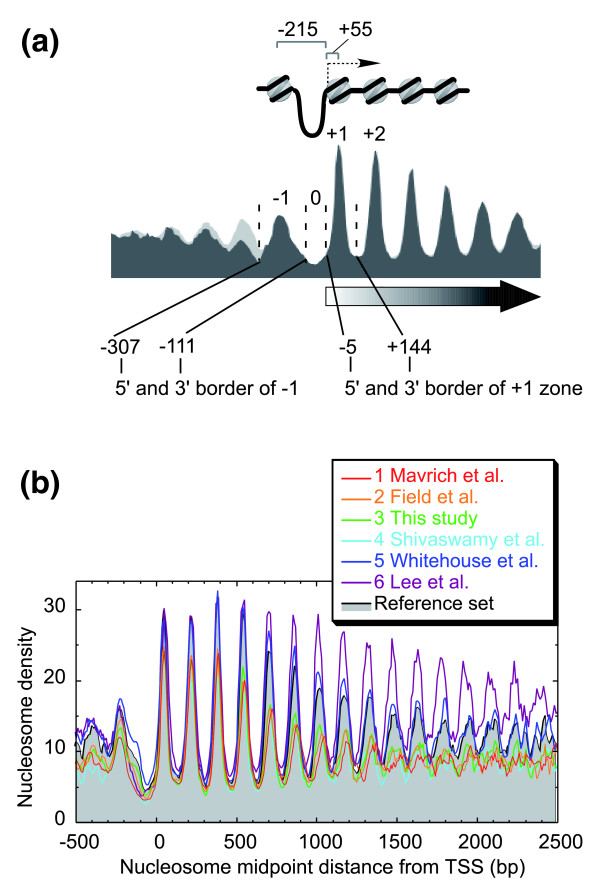
**The canonical -1, NFR, +1, +2, etc. nucleosome organization around the TSS is preserved in all datasets**. **(a) **Illustration pointing out the -1, 0, +1 zones for systematic naming of nucleosome positions. Also shown is the distance from the TSS to the -1 and +1 nucleosomes. **(b) **Distribution of nucleosome calls in each of the six datasets around the TSS. Only nucleosomes having >50% occupancy were considered. The reference set is shown as a gray-filled plot. Note that sets 5 and 6 represent hidden Markov modeling or Pearson best fit of tiling array data, and thus represent modeled positions, based upon measured periodicities. Consensus positioning at further distances from the TSS may be artificially maintained in those datasets.

The distribution of reference nucleosomes around the combined set of all mapped 7,496 RNA polymerase II TSSs displayed the expected -1, NFR, +1, +2, +3, etc. canonical arrangement, with each of the six datasets in good agreement (Figure 3b).

We also examined the distribution of reference nucleosomes around subclasses of genes, including TATA-less and TATA-containing genes, cryptic unstable transcripts (CUTs), stable untranslated transcripts (SUTs), and tRNA genes (Figure S4 in Additional data file 2), and obtained similar results as described before [11]. However, the nucleosome distribution around CUTs and SUTs has not been previously described. Their nucleosome organization is essentially the same as for other RNA polymerase II-transcribed genes, indicating that their regulatory chromatin context may be essentially the same as other RNA polymerase II-transcribed genes.

The uniformity of positioning relative to the TSS was evident out to 2 kb in all datasets, with the strongest relationship observed with TATA-less genes (Figure S4 in Additional data file 2). The apparently strong downstream positioning detected in sets 5 and 6 may be more a reflection of fitting data to an idealized pattern (set 5) or idealized positioning estimated by hidden Markov modeling (HMM; set 6) than a true measure of individual positioning (Figure 3b). Nonetheless, such idealized positions were borne out (and thus validated) in the reference data set, meaning that while downstream nucleosomes tend to lose their spacing relationship with the TSS (likely due to delocalization as discussed below), they do tend towards the expected positions.

The canonical positions in datasets 1 to 4 were less uniformly positioned relative to the TSS at position +5 and beyond (Figure 3b), suggesting that nucleosomes at positions +1, +2, +3, and +4 may be physically distinct in some way from other downstream nucleosomes.

### Nucleosome fuzziness

Previously, we and others had reported that nucleosome phasing was strongest at the +1 position [7,11]. Phasing progressively decreased towards the 3' end of genes, and nucleosomes that were not located at canonical intervals tended to be much less phased (more fuzzy) than their canonically positioned counterparts [11]. The latter observation suggested that nucleosomes that appear to be mis-positioned with respect to the TSS were positionally unstable rather than having had their location mis-identified or the TSS mis-identified. Otherwise, their fuzziness should be similar to that at nearby canonical positions. In principle, we could not rigorously exclude the possibility that the higher fuzziness of mis-positioned nucleosomes resulted from randomly distributed tags of contaminating DNA. Therefore, we re-opened this question.

Instead of using the standard deviation of tag locations around the nucleosome midpoint as a measure of fuzziness [11], we used the standard deviation of the positional calls made from each of the six datasets (that is, for each nucleosome the standard deviation was calculated for the six called positions). By using nucleosome calls, and only those having at least 50% occupancy, we essentially eliminated any interference by putative contaminating DNA.

**Figure 4:**
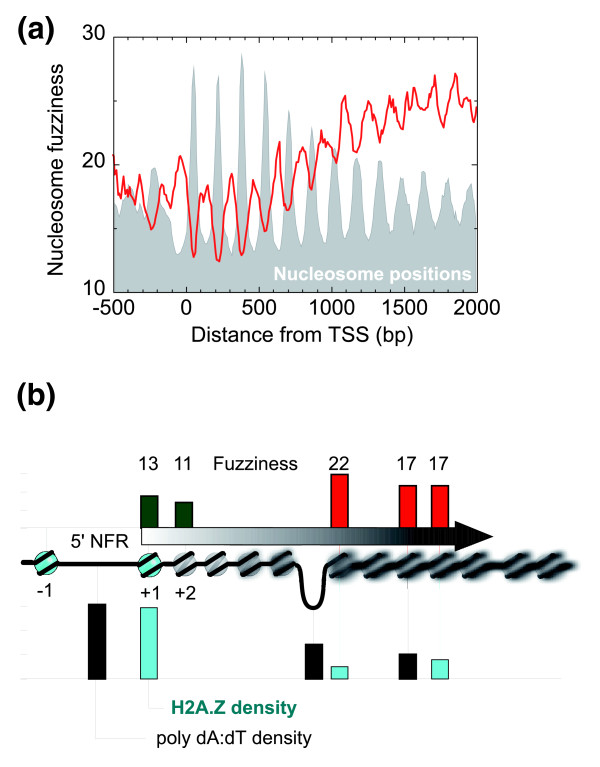
**Nucleosome fuzziness relative to TSS**. **(a) **Fuzziness is reported as the standard deviation of the six input nucleosome locations for each individual reference nucleosome. Nucleosome distances from the TSS were binned in 10-bp intervals, and the distribution smoothed using a three-bin moving average. Nucleosomes were required to have at least 50% occupancy and be called by at least four of the datasets. **(b) **Illustration of a nucleosomal array and NFRs (≥147 bp) with particular emphasis on border nucleosomes at the 5' end of genes (+1 position) in comparison with those elsewhere in the genome (that is, not at positions -1 through +4, nor at the end of genes nor in intergenic regions; nucleosomes were required to have ≥50% occupancy and be called by at least five datasets). Shown are bar graphs of quantitative measures of nucleosome fuzziness, H2A.Z/H3-H4 ratios, and poly dA:dT (A_≥5 _or T_≥5_) density in all nucleosomes or NFRs (≥147 bp) having the illustrated property (border versus non-border nucleosomes, and 5' NFR versus genic NFRs).

In agreement with prior results, not only were more TSS-distal nucleosomes more fuzzy, but reference nucleosomes that were not at their canonical locations were much more fuzzy than their counterparts at canonical distances from the TSS (as evidenced by the peaks and valleys of the red trace in Figure 4a). This further reaffirms the notion, using six independent datasets, that nucleosomes that are not at their canonical location tend to be positionally unstable and may reflect metastable nucleosome states (for example, remodeled states during transcription).

### Properties of nucleosomes that border nucleosome-free regions

The borders between arrays and NFRs are of interest because some cellular mechanism must keep nucleosomal arrays from 'spilling' into the adjacent NFR. Indeed, several studies have implicated locally bound proteins and/or poly dA:dT tracts as important for maintaining NFRs [7,11,12,17,19,20,22-24]. Although NFRs are found at the beginning and end of genes, many can be found within genes. This begs the questions as to whether such internal NFRs have the same structure as promoter NFRs, which would implicate them in internal transcription initiation. To address this possibility, we examined NFRs that were ≥147 bp to ensure that they were large enough to accommodate a nucleosome even though none was detected. We compared the fuzziness of nucleosomes at such NFR borders, and compared them to those next to promoter NFRs (that is, at +1). As shown in Figure 4b, border nucleosomes at non-+1 positions had higher levels of fuzziness (red bar graph indicated by '22') than that seen at the +1 position (green bar graph indicated by '13'). Thus, nucleosomes that border NFRs are not necessarily highly phased, as seen with promoter NFRs. Apparently, some aspect of the 5' end of genes specifically positions the +1 border nucleosome and neighboring downstream nucleosomes.

In principle, the position of a nucleosome that borders an NFR could range from highly positioned to delocalized, depending upon how diffuse the positioning element is. For example, a transcription factor bound to a specific sequence may establish well-positioned nucleosomes. However, a nucleosome exclusion sequence such as a poly dA:dT tract might vary in its exclusion potential based upon the length and base composition of the tract. As a result, a neighboring nucleosome might be presented with a 'soft' (more diffuse) border.

To address whether poly dA:dT tracts, which are linked to promoter NFRs, are also linked to non-promoter NFRs, we examined whether NFRs (≥147 bp) that were not designated as promoter 5' NFRs had an enrichment of poly dA:dT tracts compared to the rest of the genome. As shown in Figure 4b (black bars), little or no enrichment of poly dA:dT tracts was seen in genic NFRs (≥147 bp) when compared to positive (5' NFRs) and negative (genic nucleosomal) control sites. Thus, there are likely to be other mechanisms for maintaining NFRs besides the presence of poly dA:dT tracts.

NFRs that are far removed from the 5' and 3' ends of genes might represent internal promoter regions for RNA polymerase II. To address this possibility, we examined whether such NFRs contained a key hallmark of promoter nucleosomes: the replacement of H2A with the histone variant H2A.Z (histone variant Htz1). However, we found no enrichment of H2A.Z in genic nucleosomes that border NFRs (≥147 bp) compared to positive (5' NFRs) and negative (other genic nucleosomes) controls (Figure 4b, cyan bars). This implies that these NFRs within genic regions were not likely internal promoters, and is consistent with the lack of detection of TSSs in such regions.

Taken together, these analyses suggest that promoter NFRs are quite different from internal NFRs in terms of border nucleosome fuzziness and H2A.Z content, and poly dA:dT tract density. Since both types of NFRs are traversed by RNA polymerase II, it seems unlikely that transcription *per se *is a predominant determinant of such nucleosome organization. Indeed, RNA polymerase II tends to create delocalized nucleosomes [6]. Rather, some aspect of promoters, such as a combination of poly dA:dT tracts, positioning sequences, and bound factors, may play a role in establishing the canonical nucleosome organization around promoters. The higher fuzziness of nucleosomes that border genic NFRs indicates that such borders are unlikely to be generated by sequence-specific DNA binding proteins, which would be expected to produce a fixed border and highly phased border nucleosomes.

## Discussion

The ability to determine the precise locations of all nucleosomes in a genome was unimaginable ten years ago. Yet, remarkably, within the past two years, four different technology platforms (high density tiling arrays, pyrosequencing, sequencing by ligation, and sequencing by synthesis) have provided high-resolution nucleosome maps of the yeast genome. Each map, and thus each platform (Affymetrix, Roche/454, Illumina/Solexa, and Applied Biosystems), are nearly indistinguishable, reflecting a remarkable degree of concordance. The median mapping error is on the order of 5 to 7 bp genome-wide, and <5 bp for regions of highly phased nucleosomes. We suspect, therefore, that for nucleosome mapping, the technology has been perfected. What 'error' remains may largely be due to biological variation in positioning (phasing), which in many locations in the genome is nearly random, and thus defining a position is meaningless. However, strong nucleosome phasing and canonical positions exist around the start and end of genes, but even at these positions nucleosomes might occupy multiple translational settings in the context of a single rotational phase [10].

Our study, particularly the inclusion of a fully saturating depth of coverage nucleosome map, reveals that NFRs are truly devoid of nucleosomes, rather than being modestly depleted or having low but significant levels of occupancy. Because nucleosomes were covalently crosslinked *in vivo*, and only the approximately 150 bp of DNA that is crosslinked to histone H3 was immunopurified and gel purified in some of the most complete datasets, transient nucleosomes would have been detectable. However, remodeled or partial nucleosomes, in which less than approximately 120 bp of DNA was protected from MNase, might have gone undetected due to size selection of the DNA.

Other studies involving microarray hybridization of nucleosomal DNA and HMM of nucleosome positions provided estimates of >70,000 occupied nucleosomes positions. HMM uses a training set of well-defined positions to provide estimates of positions throughout the genome. Consequently, training on uniformly spaced positions may cause such spacing to be perpetuated at regions where spacing is less defined or occupancy is negligible. As such, we suspect that HMM may over-estimate the uniformity and density of nucleosomes in a genome, although our studies with other datasets validate the HMM approach as identifying the 'best' positions, should they become occupied or phased.

Knowing where nucleosomes reside is key to understanding how access to DNA sequences is controlled and ultimately how transcription, DNA replication, recombination, and repair are controlled. Gene activation and repression are accompanied by loss and gains of nucleosomes, respectively [13]. Chromatin remodeling complexes will reposition nucleosomes to mitigate cryptic TSSs [9]. Given the location of the -1 nucleosome in the neighborhood of the upstream activating sequences, and the +1 nucleosome encroachment on the TSS, it is becoming clear that individual nucleosomes will have specific functions [6]. Therefore, a standard and facile referencing system is helpful for identifying the most accurate position of every nucleosome and providing a consistent numbering system.

While the reference set of nucleosomes presented here might provide a useful resource for systematically identifying corresponding nucleosomes in orthologous experiments, it does not supplant the need for producing a *de novo *reference dataset in a set of related experiments. Such a *de novo *reference state might, for biological or technical reasons, be distinct at some loci from the reference state generated here.

Our reference system numbers nucleosomes with respect to the TSS, starting with the 0 position, which represents the canonical 5' NFR. Although generally nucleosome-free, the 5' NFR may be occupied by a nucleosome at some repressed genes (for example, *PHO5 *and *RNR3*). The referencing system proposed here is inconsistent with the historical numbering system used to study several of these model genes because those genes lacked an NFR and upstream nucleosome numbering thus began with -1. However, most genes have 5' NFRs, and such nucleosome exclusion is typically hard-coded into the DNA [23,25]. Therefore, we feel that it would be less confusing to start the numbering at '0', to reflect this unique nucleosome-free property. Nucleosomes residing at the '0' position are likely, therefore, to represent a minority of inducible genes that are repressed by placement of a nucleosome over the core promoter.

Since individual nucleosome positions such as +1 versus +2 may have distinct functions based upon distance from the TSS, we chose to ensure that the numbering system preserved the canonical zones in which nucleosomes appear. Thus, the first nucleosome downstream of the TSS is normally called +1. However, if the first downstream nucleosome is found in a region where the +2 nucleosome normally resides, then it is numbered as +2 instead of as +1.

In yeast, as in some metazoans such as flies and worms, genes are so tightly packed that a nucleosome may 'belong' to two different genes. Our numbering system assigns both gene-specific numbers to the same nucleosome. Thus, the full complement of yeast nucleosomes can be filtered to acquire nucleosomes of specific positional characteristics.

The methods used here for numbering nucleosomes and defining a reference position should be applicable to any eukaryotic genome, once sufficient high quality and complete nucleosomal datasets are available. Moreover, this report may be the first such description of a systematic means of identifying 'soft' features in the genome. The use of the term 'soft' for protein-DNA interactions reflects the fact the such interactions are experimentally determined rather than computationally predicted, and may shift from one experiment or condition to another.

## Materials and methods

### Nucleosome data sets

Six independent nucleosome datasets from *S. cerevisiae *strain S288C or its BY4741 derivative were used (summarized in Table 1; Additional data file 1). Five were from previously published datasets, and one using the SOLiD platform is presented here (set 3). Sets 1 to 4 employed DNA sequencing to identify individual nucleosomes, and consensus positions were estimated from clusters of sequencing reads or tags. Our newly generated set 3 contained nearly ten times the number of tags as all other sets combined.

For dataset 3, nucleosome preparations were made from a BY4741 strain containing a carboxy-terminal TAP tag on histone H3. Details for MNase digestion, H3 immunoprecipitation, and gel purification are described elsewhere [10]. The amplified mono-nucleosomal DNA was sequenced using SOLiD. The SAT software tool accompanying SOLiD was used to map tags to the yeast reference genome. Only uniquely matched tags with up to three mismatches out of 36 bp were used to predict nucleosomes.

For datasets 1 and 3, the 5' end of each read was considered to be an independent measure of one border of a nucleosome. In all cases, the goal was to identify the nucleosome midpoints and so 73 bp was added to each read that mapped to the plus strand, and 73 bp was subtracted from each read that mapped to the minus strand. The reads used to predict nucleosomes in dataset 2 have a length of 127 to 177 bp [12], which spans the entire measured nucleosome and thus simultaneously identifies both nucleosome borders. The midpoint of these reads was treated as the nucleosome midpoint.

The nucleosome prediction program GeneTrack was employed to make nucleosome consensus calls based on these midpoints as was done in previous studies [10,11,14,26] (Figure 1a). Each mapped read/tag was replaced by a probability function (having a sigma value = 20) that a measured 'call' is located within a certain distance of the putative nucleosome midpoint. GeneTrack then generated a smoothed probability landscape of nucleosome locations throughout the genome by summing the probability function over all reads. GeneTrack makes coarse-grain calls by identifying the highest peaks (in order of peak height) as consensus nucleosome midpoints and setting up an exclusion zone (in this case 147 bp, corresponding to the expected length of nucleosomal DNA) centered over the peak such that no new nucleosome peaks may be called within that exclusion zone.

Datasets 4 to 6 used nucleosome calls as made by the authors of those studies. In brief, a Parzen window-based approach was employed to predict the borders of a nucleosome and then infer the nucleosome midpoint in dataset 4 [13]. Nucleosome calls in dataset 5 iteratively fit the probe signal of the idealized nucleosomes to the tiling array probes. The probe position with the best fit (that is, the highest Pearson correlation coefficient) was defined as the nucleosome midpoint [9]. Nucleosome calls in dataset 6 used the probes in several characterized key loci as the training data and applied HMM to predict nucleosome positions [8]. In as much as the latter two methods assume regular nucleosome arrays even at loci where such regularity may not exist, such methods may overestimate the number of actual nucleosomes in the genome and create a more idealized rather than actual pattern.

### Determination of a measured 'reference set' of nucleosome positions

Consensus nucleosome midpoint positions were combined from each of the six datasets and used by GeneTrack to make a new consensus, which we define as the measured 'reference set' of positions. A total of 61,110 measured reference nucleosomes were determined (59,915 at ≥5% occupancy). We assigned 5,043 non-overlapping regions that were at least 147 bp and lacked any measured nucleosome as 'hypothetical' nucleosome placeholders (Additional data file 1; is also described in more detail below), which, under other growth conditions, might be occupied by nucleosomes.

### Assigning individual reference nucleosomes a numerical position relative to the TSS

#### Overview

Initially we sought to number each nucleosome according to its location within well-defined consensus zones of where nucleosomes tend to reside relative to the TSS (for example, see Figure 3a). These zones were spaced in 165-bp intervals, corresponding to the canonical nucleosome spacing. However, in some cases, close packing resulted in more than one nucleosome in a zone, which thus acquired the same positional number. Thus, we opted for a more complex scheme in which nucleosome positions in the -1 and +1 zones (where the numbering scheme originates) were first identified (see below). Next, adjacent nucleosomes were numbered sequentially. When a linker of ≥147 bp was encountered, one or more hypothetical nucleosomes were inserted, as dictated by the size of the linker. We did this because under another cellular state such regions may become occupied by nucleosomes. These hypothetical nucleosomes are listed under a separate tab in Additional data file 1. The numbering continued, utilizing the hypothetical positions, until the end of the gene was reached. A nucleosome could be assigned more than one positional number if more than one TSS was used in assigning a position (for example, a nucleosome may be assigned to the +1 position for one gene, and to a -1 position for an adjacent divergently transcribed gene).

#### Demarcation of the -1, 0, and +1 zones

The canonical -1, 'NFR', +1 nucleosome arrangement around the vast majority of yeast TSSs is conserved in all the datasets. Therefore, we used this canonical nucleosome distribution pattern around the TSS to demarcate -1, 0, and +1 nucleosome zones. The valley minimum between the +1 and +2 nucleosomes demarcated the 3' border of the +1 nucleosome zone (Figure 3a). The same level of nucleosome occupancy on the 5' side demarcated the 5' border of the +1 nucleosome zone. Similarly, the valley minimum between -2 and -1 nucleosomes demarcated the 5' border of -1 nucleosome zone, and the same level of nucleosome occupancy demarcated the 3' border of the -1 zone. The -1 and +1 nucleosomes bracket a consensus NFR. Thus, we obtained three definable zones relative to the TSS to which a nucleosome midpoint may be classified: -1 (from -307 to -111), 0 (from -110 to -6), and +1 (from -5 to +144). The canonical (peak) distance from the TSS to the midpoint of the -1 nucleosome is -215 bp, and +55 bp for the +1 nucleosome. These zones and the peak distance relative to the TSS were used for labeling nucleosomes in this study.

#### Insertion of hypothetical nucleosomes

We found 4,628 linkers (defined in this instance as the distance from one measured reference nucleosome border to the next adjacent measured reference nucleosome border) of size ≥147 bp. We inserted evenly spaced hypothetical nucleosomes in these regions until no more sequence ≥147 bp existed. This resulted in a total of 5,043 potential or hypothetical nucleosomes inserted, resulting in a total of 66,153 measured plus hypothetical nucleosome positions that serve as the reference set of nucleosome positions. The coordinates of the potential nucleosomes are listed under two separate tabs in Additional data file 1.

#### Labeling individual reference nucleosomes

Each reference nucleosome was numbered according to its midpoint/dyad distance from the TSS according to the following rules. Any reference nucleosome (including both measured and hypothetical) whose midpoint was located within the zones -1, 0, or +1 was labeled as such. For a given gene, we set *D*_*i *_to denote the distance of the *i*^th ^nucleosome midpoint from TSS, and *N*_*i *_to denote its numerical position relative to the TSS. Therefore, *N*_1 _equals +1. In some cases where no nucleosome was present in the +1 nucleosome zone because the nearest nucleosome midpoints were just outside the +1 border, *D*_1 _was set to the default value of +55 bp, which is the distance from the TSS to the peak coordinate of the +1 consensus nucleosome. The number of nucleosomes that can be placed in the region between the midpoints of the adjacent nucleosomes was set to (*D*_*i *_- *D*_*i*-1_)/165, whose nearest integer we denote as *I*_*i*_. Note that '165' refers to the nucleosomal core DNA length (147 bp) plus linker (18 bp). The numerical position of the *i*^th ^nucleosome relative to the TSS is *N*_*i*-1 _+ *I*_*i*_. In this way, the reference genic nucleosomes were designated as +1, +2, and so on. The last reference nucleosome midpoint located within 50 bp downstream of and closest to the transcription termination site (TTS; equivalent to the polyA addition site) was defined as the terminal nucleosome to its associated gene. An asterisk was appended to its label as a postfix. Intergenic nucleosomes that were not assigned a position label were left blank. All reference nucleosomes were systematically named using their midpoint chromosomal coordinates prefixed with a character 'N' (consensus measured nucleosomes) or 'P' (potential nucleosome): for example, N1:192 represents a measured nucleosome on chromosome 1 having a midpoint coordinate at 192.

### Occupancy level of reference nucleosomes

For many analyses, the occupancy level of individual nucleosomes is a useful metric. We chose to utilize only the sequencing datasets (1 to 4) to provide a measure of occupancy level. The read or tag count per nucleosome was first normalized to the modal value for the entire dataset. This normalization makes the assumption that the most frequent tag count corresponds to nucleosomes that fully occupy their position, and is justified by the reasonable expectation that the most commonly observed occupancy level of a nucleosome would be 100% (a site always being occupied), inasmuch as chromosomes must always have the bulk of their DNA charges neutralized. Thus, as a practical matter, such normalized occupancy levels that are >100% are re-coded as 100%.

Since nonspecific DNA can contaminate nucleosome preparations, we did not want to assign nucleosome occupancy levels to NFRs due to contamination. As evident in Figure S1 in Additional data file 2, the high coverage of dataset 3 results in a statistically high number of nucleosomes that have a very low tag count (see the deviation of the trace from the expected normal distribution at tag counts <20). This small deviation may represent contamination. We calculated the standard deviation (σ) of the tag distribution shown in Figure S1 in Additional data file 2. If the tag count for a nucleosome fell below the overall mode value minus 2σ (for example, tag count <37 for set 3), then its occupancy level was set to zero. All remaining nucleosomal tag counts between the mode minus 2σ and the mode were scaled between 1 and 99%. The normalized occupancy was calculated for other datasets in a similar manner. These normalized occupancy values are presented in Additional data file 1. The mean of these values across datasets 1 to 4 were recorded as the occupancy level for the reference nucleosome (see column 8 'occ' in Additional data file 1).

#### Determination and classification of linkers and nucleosome-free regions

Unless indicated otherwise, a linker is defined here as the distance from one measured reference nucleosome border to the next adjacent measured reference nucleosome border, in which each measured nucleosome has an occupancy level of >5%. All such linkers in the genome were identified from the reference set. If a linker was >79 bp (corresponding to the minima between the bimodal distribution of linker lengths shown in Figure 1f), it was named 'NFR'. All others retained the name 'linker'. The linker/NFR that overlapped or was the closest to the canonical location of the 5' NFR position for RNA polymerase II-transcribed genes (58 bp upstream of the TSS; Figure 4b) was designated as the 5' NFR or 5' linker for the associated TSS. The same was done for TTS for 3' NFRs/linkers. All other linkers/NFRs located between a 5' and a 3' linker/NFR were designated as genic linkers/NFRs. The same was done for all other identified genomic features, assigning the closest linker/NFR to the feature start and end coordinate (Additional data file 1).

### Fuzziness of reference nucleosomes

Fuzziness is considered to be the opposite of phasing. That is, fuzziness is the extent to which a nucleosome is delocalized at a position. Previously, we quantified fuzziness by reporting the standard deviation of tag distances from the consensus position. Here we report the fuzziness of each reference nucleosome as the standard deviation of distances of each consensus nucleosome in a dataset from the reference position. That is, a maximum of six consensus distances were used to compute the fuzziness call of a reference nucleosome (Additional data file 1).

### Distribution of nucleosomes around the transcription start site

TSSs were retrieved from the *Saccharomyces *Genome Database [27], and combined with novel transcripts, anti-sense transcripts, SUTs, and CUTs from published work [28,29] after removal of the redundant transcripts (Additional data file 1). The method for plotting the distribution of six sets of input nucleosomes and the newly derived reference set around the TSS was described previously [11,14]. In brief, nucleosomes were aggregated over the genome into individual 10-bp bins determined by the nucleosome midpoint distance from the TSS. Consecutive bin counts were smoothed using a three-bin moving average. Nucleosomes located at less than 300 bp internal or external to a TSS or TTS of a nearby gene were removed from the analysis to minimize potential influence from nearby genes. For short genes or overlapped genes, a minimum 300-bp region flanking the TSS was analyzed. The nucleosome count was normalized to gene number in each bin.

### Assigning newly measured nucleosome positions to a reference nucleosome position

As additional nucleosomal datasets are collected under different cellular conditions, new insights may be best attained by comparing each newly identified nucleosome position to its reference position and/or coordinate. To do this we have written a script to identify the closest reference nucleosome to each measured nucleosome. The measured nucleosome then acquires the profile of the reference nucleosome, such as the associated genes and the corresponding positional number relative to the TSS. Such a service is available via at the Penn State Genome Cartography website [21].

### A retrieval system for reference nucleosomes

We built a retrieval system [21] to allow users to access the reference nucleosome positions for any gene(s) in two ways: via a browser query for a gene name or chromosomal coordinate; or via a text query that produces a text file of nucleosomes for a gene or chromosomal coordinate, including its numerical position and its distance from the TSS.

## Abbreviations

CUT: cryptic unstable transcript; H2A.Z: histone variant Htz1; HMM: hidden Markov modeling; NFR: nucleosome-free region; SUT: stable untranslated transcript; TSS: transcription start site; TTS: transcription termination site; YPD: yeast peptone dextrose.

## Authors' contributions

BFP conceived of the study. CJ performed the analysis, computation, and software development. CJ and BFP wrote the manuscript.

## Additional data files

The following additional data are available with the online version of this paper: an Excel table compilation of (by tabs) nucleosome positions, hypothetical nucleosomes, arrays, genes, and linkers/NFRs (Additional data file 1); supplementary Figures S1, S2, S3 and S4 (Additional data file 2).

## Supplementary Material

Additional data file 1 Nucleosome positions, hypothetical nucleosomes, arrays, genes, and linkers/NFRs. Click here for file

Additional data file 2 Figures S1, S2, S3 and S4. Click here for file
