# Supplementary material for: A compiled and systematic reference map of nucleosome positions across the Saccharomyces cerevisiae genome
Source: Genome Biol. 2009 Oct 8;10(10):R109. doi: 10.1186/gb-2009-10-10-r109 (PMC2784324; doi:10.1186/gb-2009-10-10-r109)
Supplement: Additional data file 2 — Figures S1, S2, S3 and S4. [file gb-2009-10-10-r109-S2.PDF]

Additional data file 2

Jiang and Pugh, Genome Biology 2009

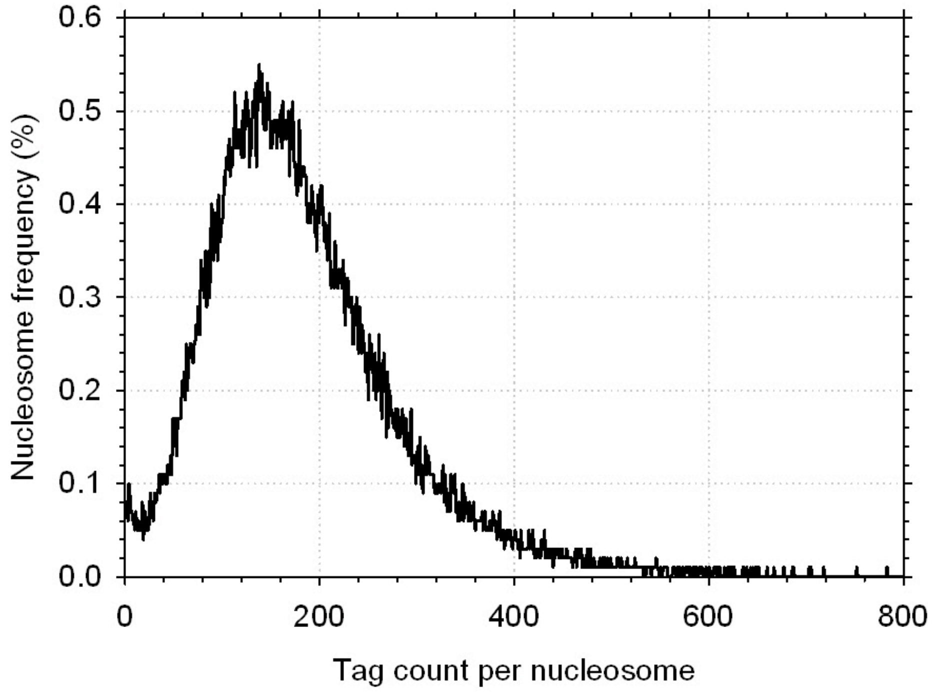

Figure S1. Frequency distribution of tag counts per nucleosome for the sequencing dataset 3 (SOLiD). The most frequently encountered tag count was 150 tags per nucleosome. The tag count per nucleosome at the peak of the distribution was defined as the mode. A normalized nucleosome tag count relative to this mode value was assigned to each nucleosome and provides a measure of nucleosome occupancy (see Methods explanation). Values higher than the mode were re-coded to 100% occupancy, and are assumed to reflect statistical variation. Values lower than the mode minus 2 standard deviations (<37 tags) were set to zero occupancy.

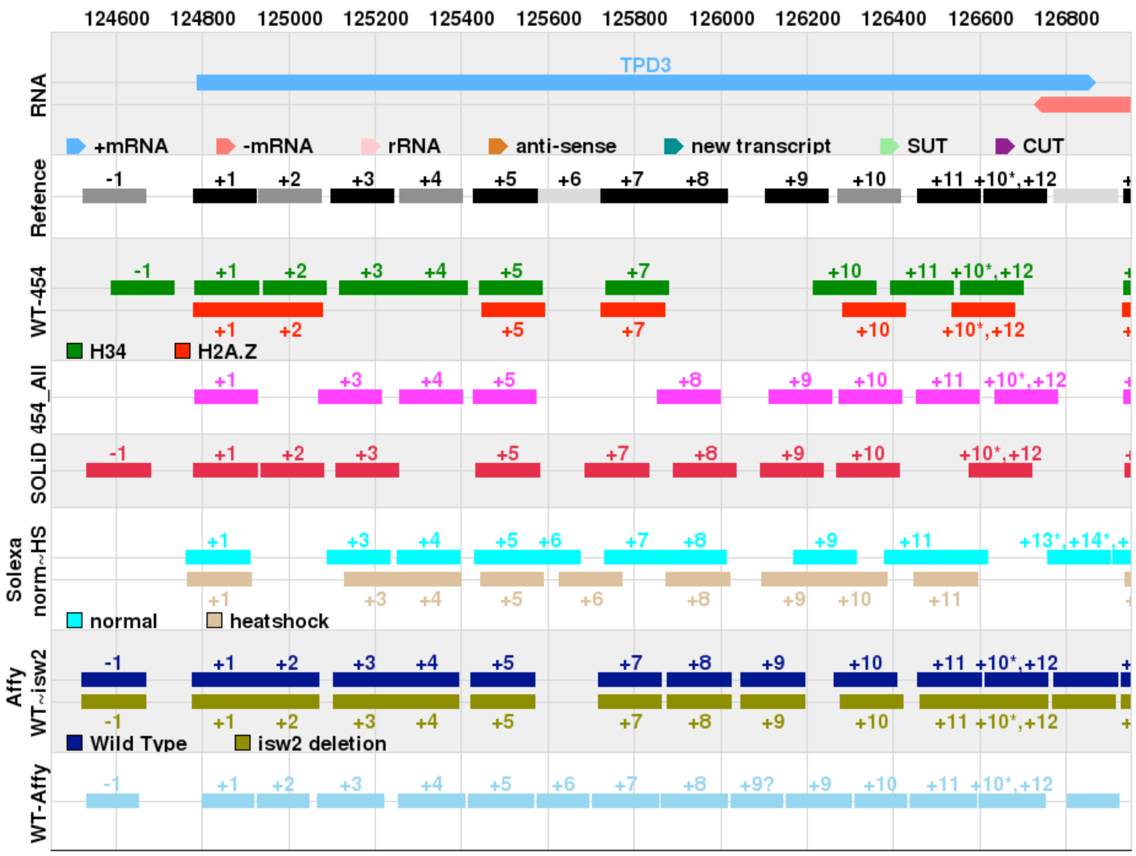

Figure S2. Browser screen shot showing inconsistency between the six sets of input nucleosomes at gene TPD3. In Reference track, the color of the box indicates the mode-normalized nucleosomal occupancy: light gray: < 5% (i.e., in NFRs), intermediate gray: 5-50% (i.e., in NDRs), dark gray: 50-100%, black: 100%.

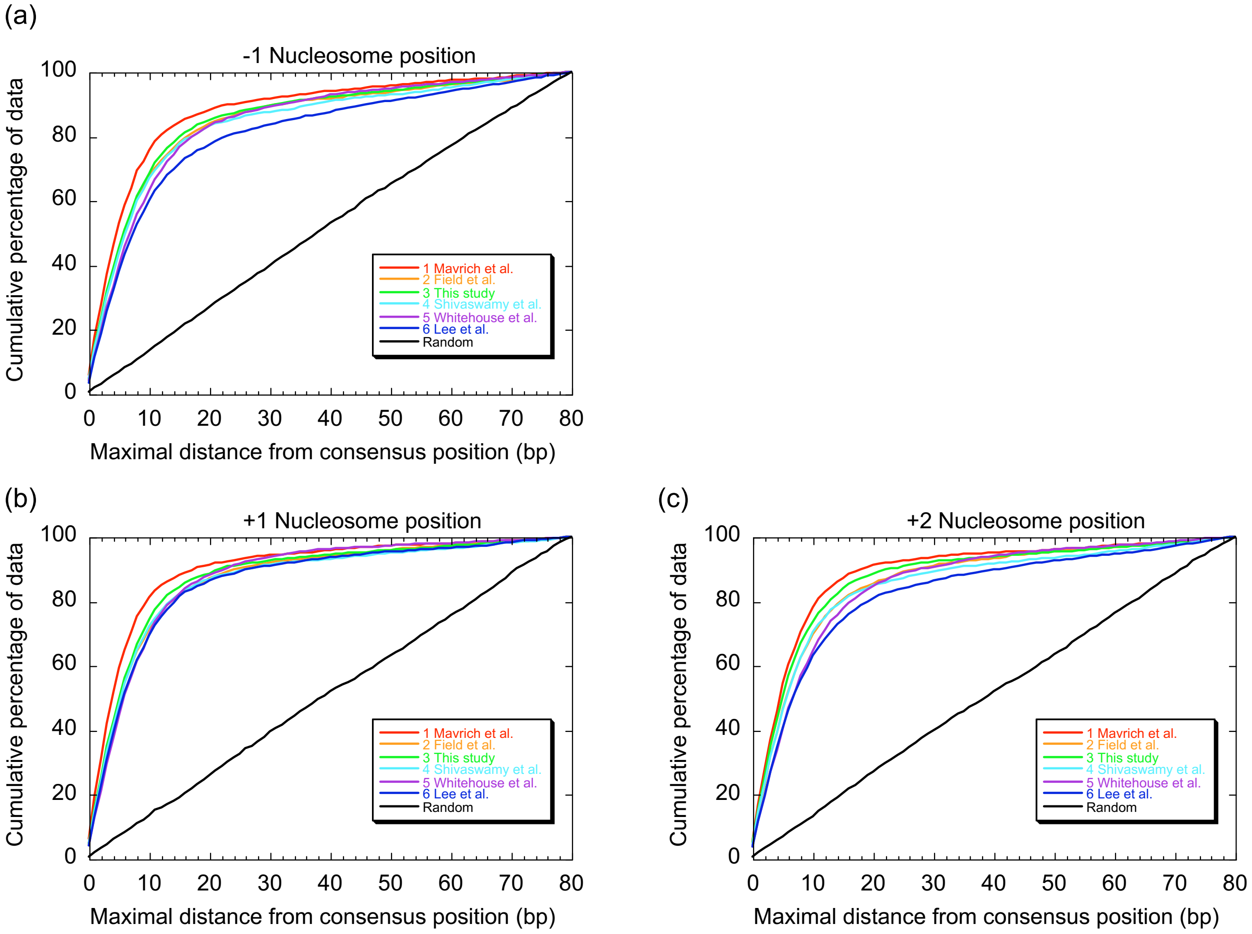

Figure S3. Cumulative error associated with the six sets of input nucleosomes and random nucleosomes. Error interval is the midpoint distance between the reference nucleosome and the query nucleosomes. **(a)** +1 nucleosomes; **(b)** +2 nucleosomes; **(c)** -1 nucleosomes. Each dataset is described in Table 1.

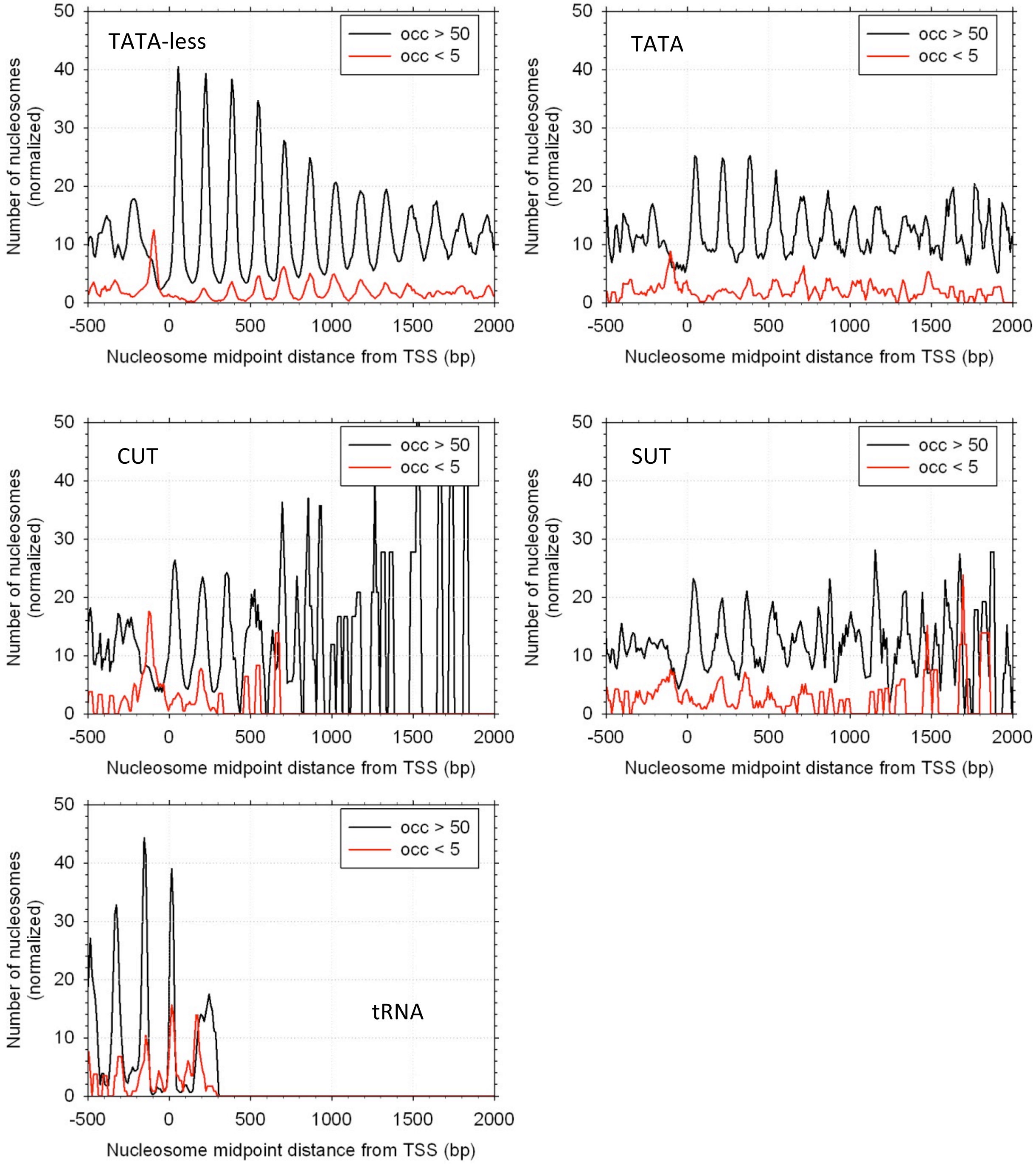

Figure S4. Distribution profile of measured reference nucleosomes around TSS of different feature types. "occ > 50" denotes occupancy levels of nucleosomes were required to be >50%. "occ < 5" is a map of NFRs (≥147 bp).
